# Characteristics of Human Babesiosis in Europe

**DOI:** 10.3390/pathogens12020323

**Published:** 2023-02-15

**Authors:** Anke Hildebrandt, Jeremy Gray, Estrella Montero

**Affiliations:** 1 Department of Internal Medicine I, St. Vincenz Hospital Datteln, 45711 Datteln, Germany; 2 Institute of Medical Microbiology, University Hospital Münster, 48149 Münster, Germany; 3 UCD School of Biology and Environmental Science, University College Dublin, D04 N2E5 Dublin, Ireland; 4 Parasitology Reference and Research Laboratory, Centro Nacional de Microbiología, Instituto de Salud Carlos III, Majadahonda, 28220 Madrid, Spain

One of the Editor’s choice articles in 2021 published in *Pathogens* was a review of human babesiosis in Europe [1]. Babesiosis is an emerging tick-borne disease caused by intraerythrocytic protozoa that are primarily transmitted by hard-bodied (ixodid) ticks and rarely perinatally, through blood transfusion and organ transplantation [2,3]. More than 100 *Babesia* species infect a wide spectrum of wild and domestic animals worldwide and six have been identified as human pathogens [2,4]. European human babesiosis emerged in the 1950s and more than 60 cases have now been published, the majority of which present as life-threatening fulminant infections. So far, three European *Babesia* species have been confirmed as human pathogens: *B. divergens*, *B. venatorum* (*Babesia* sensu stricto (s.s.) group) and *B. microti* (*Babesia* sensu lato group) [5,6]. 

Cattle are regarded as the reservoir hosts for *B. divergens* infections, which has been supported by 18S rRNA gene sequencing [7]. It has frequently been suggested that certain deer species may also serve as a source for human *B. divergens* infections [8], but so far there is no convincing evidence for the thesis of deer as reservoir hosts. The reservoir host for *B. venatorum* in Europe is the roe deer (*Capreolus capreolus*) [1], but in China, where approximately 50 cases have been diagnosed [9], the suspected reservoir host is the sika deer (*Cervus nippon*) and the probable vector is *Ixodes persulcatus* [10]. *Babesia microti* is a complex species that mainly infects small mammals and consists of five clades [11]. Parasites from Clade 1 (US type) cause most cases of human babesiosis worldwide [11]. Another *B. microti* strain, the Munich-type, belongs to Clade III and is widely distributed in Europe [12]. Originally, it was not thought to be zoonotic, but the DNA of this parasite has now been detected in seven patients in Europe, all of which were asymptomatic or had non-specific symptoms [13,14]. The zoonotic potential of this piroplasm is still uncertain.

The castor bean tick, *Ixodes ricinus*, is thought to be the vector of all European zoonotic *Babesia* spp. [1], though there is no definitive evidence that the *B. microti* Munich strain can be transmitted by this tick species. *Ixodes trianguliceps* seems to be the most important vector of the Munich strain [15]. This strain has also been detected in *I. ricinus* ticks in Europe [16,17,18], but this does not prove vector competence and the enzootic cycle in nature is not yet fully understood. Most of the *B. microti* cases in Europe appear to have been caused by Clade 1 (US-type) parasites, which have been shown in the laboratory to be transmitted by *I. ricinus* [11]. To date, no definitive molecular sequencing of field-derived *I. trianguliceps* has detected the US-type genotype in this tick species [11]. 

*Babesia* spp. parasitise erythrocytes and the spleen plays a central role in blood defence by clearing infected erythrocytes from the bloodstream and mounting protective immune responses [1,19,20]. Isolation of *Babesia* spp. from human blood samples is important for further investigation, but unfortunately in most clinical cases this has not been attempted [1]. 

European human babesiosis was first reported 1957 and concerned a fatal case that occurred in the former Yugoslavia, now Croatia, where a splenectomised part-time farmer with fever and severe haemoglobinuria died 10 days after symptoms first appeared [21]. Many more cases have since been reported, mostly occurring in France and the British Isles, and have usually been fulminant infections in splenectomised patients, although a few patients were young and immunocompetent. At least 35 were caused by *B. divergens,* 5 by *B. venatorum* and 11 by *B. microti* (excluding imported cases) [1].

Hildebrandt et al. [1] have collated all published cases between 1998 and 2021, with information on country, age, course of disease, comorbidity, and diagnostic and therapeutic aspects. Thirteen reported cases of human babesiosis in Europe were imported, all caused by *B. microti* with travel history to the Americas. Additionally, there have been reports of ambiguous human babesiosis cases (most of them with unidentified *Babesia* spp.) diagnosed with unsatisfactory criteria or lack of clarity [1]. 

Infections transmitted by transfusion are rare in Europe, which contrasts with the USA, where they occur quite frequently [22]. So far, only one such case has been reported in Europe [23]. However, according to the cases listed by Hildebrandt et al. [1], there is increasing evidence for mild and asymptomatic infections with *Babesia* parasites. Furthermore, *B. divergens* parasites are robust enough to survive for days in blood bags and in platelet concentrates stored under blood bank conditions and produce a high final parasitaemia in vitro [24,25]. Nevertheless, transfusion transmission rarely results in babesiosis in Europe despite the frequency of the procedure [1]. At present, the reasons are uncertain. Whereas in the USA blood products are routinely screened for *Babesia* parasites [22], this does not occur in European countries, where splenectomy followed by exposure to *I. ricinus* ticks (patients may not be aware of a tick bite) are the greatest risk factors for babesiosis. General pre-disposing factors associated with higher risk of symptomatic infection and more severe illness, besides splenectomy, are impaired cellular and/or humoral immunity and advanced age [26,27].

In Europe, misdiagnosis and lack of awareness of the existence of the disease have occasionally led to delayed diagnosis, resulting in prolonged and potentially life-threatening disease. In some cases, human babesiosis was only diagnosed post-mortem. Typical clinical symptoms (e.g. fever, chills, headache, arthromyalgia, and elevated liver enzymes), in combination with a positive Coombs test, haemolytic anaemia, and elevated procalcitonin levels, should prompt further diagnostic testing [1]. 

Microscopy and PCR are useful tools for the diagnosis of acute disease. The three parasites are distinguishable morphologically in Giemsa- or Romanowsky-stained blood smears, but only by experienced diagnostic microscopists because they share important features [1,6]. In the case of PCR diagnosis, sizeable DNA fragments of the cytochrome c oxidase subunit I (COI) and 18S rRNA genes should be sequenced [1,28]. Serology is neither sensitive nor specific and cannot reliably distinguish current and past infections [6,29,30]. It is not the diagnostic method of choice in acute disease because of the time required for an antibody response to develop, which may be 2–3 weeks or possibly longer in immunocompromised patients [31]. *Babesia microti* can be distinguished serologically from *B. divergens* and *B. venatorum*, but these two species are antigenically similar [32]. Culturing, either in vivo or in vitro, is valuable for diagnostic confirmation and identification of the *Babesia* species involved, but is impracticable in routine laboratories (due to a labour-intensive and time-consuming process, availability, ethics, and sensitivity). Isolation of the parasite is especially challenging and often impossible if patients have already been treated for babesiosis. Unfortunately, no standardized PCR protocols and serology tests are available in Europe so far [1].

Several drugs have proved effective against human babesiosis alone or in combination, such as quinine, clindamycin, atovaquone, and azithromycin. In case of relapse and persistence of infection, monitoring of parasitaemia by blood smear examination and PCR is important. The review by Hildebrandt et al. [1] gives an overview of doses of antibabesial drugs in adults and children. Additionally, commonly used drug combinations and treatment alternatives for human babesiosis are discussed with regard to the parasite species and severity of the disease. In severe disease, defined according to White et al. 1998 [33] (parasitaemia > 4%, alkaline phosphatase > 125 U/L, and white blood cell counts > 5 × 10^9^/L), the combination of clindamycin and quinine is recommended. However, this treatment regimen is frequently associated with serious side effects, mainly from quinine, such as hearing loss, vertigo, and tinnitus. In some cases, these side effects can be so severe that dose reduction or discontinuation of treatment is required [34]. Exchange transfusion is recommended for severe *B. microti* infections with parasitaemia > 10% and/or organ dysfunction and all emergencies of *B. divergens* infections, provided the patient’s condition permits it [6,35]. Atovaquone is effective in clearing the infection but has low bioavailability. Therefore, higher doses, longer treatment duration, and, in some cases, the combination of atovaquone with intravenous administration of azithromycin is required [35]. The use of atovaquone, together with either azithromycin or proguanil is strongly recommended for all cases of babesiosis, especially following initial use of quinine when *B. divergens* is involved [1,6,31]. These drug combinations have been used in at least four recent cases, following problems with toxicity or inadequate efficacy of other drug regimens [31,36,37,38], and resulted in patient recovery for three of them [1,31,36,37]. The combination of atovaquone and proguanil is available as Malarone, which is marketed for malaria treatment and has already been used effectively in human babesiosis [1,14,36]. It is also worth noting that the use of immunosuppressive agents, such as Rituximab, to treat prior illnesses (B-cell lymphoid malignancies, rheumatoid arthritis, etc.) may lead to babesiosis relapse and extended persistence of *Babesia* parasites [34,39]. 

## Future Research

It is almost 70 years since the first case of human babesiosis was recorded in Europe and, as discussed above, much knowledge is now available on the nature of the disease, the parasite, and its vector. However, as also indicated above, there are still areas concerning parasite identity, vector transmission, host reservoirs, risk assessment, and case management for which information is unavailable or equivocal, and these topics should be the focus of future research. The accuracy of parasite identity is fundamental to an understanding of *Babesia* biology but is heavily dependent on detection of 18S rDNA and, although more attention is now given to other gene targets such as cytochrome c oxidase subunit I (COI) and beta-tubulin, increased focus on accurate molecular recognition of parasite genotypes is highly desirable. Accurate determination of the identity and occurrence of the widespread Munich strain of *B. microti* is necessary because, although previously thought non-zoonotic, it has recently been reported in mild and asymptomatic human infections. Its DNA has been detected in *I. ricinus* on two occasions in Poland [16,17], but the role of this tick species as the vector is still uncertain and transmission studies, as for European strains of the US genotype [40], should be undertaken. The reservoir status of red deer (*C. elaphus*) for *B. divergens* is also uncertain and the frequent citings of this host as a reservoir are entirely based on the occasional detection of *B. divergens*-like DNA in spleen or blood samples. So far, the few in vivo transmission studies carried out have not indicated that cross-infection can occur between deer and cattle. However, in the future, the ready availability of *B. divergens* blood cultures may provide the opportunity to examine the suitability of deer erythrocytes for cattle-derived *B. divergens*. Another area of uncertainty is the risk of transfusion transmission in Europe. Zoonotic *B. microti* seems to be rare, and *B. divergens* and *B. venatorum,* which usually cause rapidly fulminant infections in immunocompromised patients, were thought, until recently, to be insufficiently persistent to constitute a transfusion threat. However, there is growing evidence that mild, even subclinical, infections of these parasites may occur [1] and there could be a case for devising blood screening protocols similar to those in the USA [22]. 

The management of acute cases of babesiosis has improved considerably, especially with swifter diagnosis and the use of exchange transfusions, but the antibabesial drugs available are not very satisfactory. For example, the rapid action of quinine is offset by its toxicity. The action of atovaquone in fulminant infections with high parasitaemia is generally considered to be too slow, although in at least one study of a series of cases in the USA it was found to perform as well (in combination with azithromycin) as the combination of quinine and clindamycin [41]. Atovaquone is particularly effective against *B. divergens* [34,41] and its use as a second line treatment to ensure parasite clearance in cases caused by *Babesia* s.s. should be promoted. New antibabesial drugs are needed but current research on this topic is limited and tends to focus on infections in livestock. However, this is likely to be the best source of new effective drugs for the treatment of human babesiosis.

With increasing awareness of human babesiosis in Europe, new discoveries and questions are continuously arising. For example, a *Babesia crassa*-like parasite in human infections in Slovenia [42] and France [43] has recently emerged. *Babesia crassa* is a sheep parasite originally described in sheep in Iran [44], and has been phylogenetically characterized [45]. Human infections with *B. crassa*-like parasites, all with mild to moderate clinical symptoms, have been described in China, along with numerous isolations from ticks and sheep [46]. The One Health approach will greatly facilitate the detection, characterization, and control of such infectious threats in the future. 
